# A preliminary study to evaluate the impact of pharmaceutical care services on clinical outcome and medication adherence in type 2 diabetes mellitus patients from Ethiopian perspective

**DOI:** 10.4314/ahs.v22i4.14

**Published:** 2022-12

**Authors:** Bruke Berhanu Billoro, Abdikarem Mohamed Abdi, Wondimu Assefa Abero, Abdi Bedassa Fite, Bilgen Basgut

**Affiliations:** 1 Department of Clinical Pharmacy, Faculty of Pharmacy, Near East University, Nicosia, Northern Cyprus, Mersin10, Turkey; 2 Department of Pharmacy, College of Medicine and Health Sciences, Wachemo University, Hosanna, Ethiopia; 3 Department of Clinical Pharmacy, Faculty of Pharmacy, Yeditepe University, Istanbul, Turkey; 4 Department of Internal medicine, Wachemo University Nigist Elleni Mohamed Memorial Comprehensive Specialized Hospital, Ethiopia; 5 Department of Internal medicine, Arsi University Asella Teaching and Referral Hospital, Ethiopia; 6 Department of Pharmacology, Faculty of Pharmacy, Baskent University, Ankara, Turkey

**Keywords:** Clinical pharmacy, Ethiopia, Medication adherence, pharmaceutical care services, T2DM, Wachemo University

## Abstract

**Background:**

The role of clinical pharmacist in hospital settings of Ethiopia is still new and infant.

**Objective:**

To evaluate the impact of pharmaceutical care on clinical outcome and medication adherence in type 2 diabetes mellitus (T2DM) patients.

**Methods:**

A single cantered, pre-post interventional study design was carried out by enrolling 100 uncontrolled T2DM patients from March 1-August 30, 2020. The intervention package included assessment of pharmacological and non-pharmacological needs, counselling patients in person at the clinic, and providing educational materials.

**Results:**

Of the 100 patients initially enrolled, 87(87%) completed the follow-up and included in the final data analysis. The intervention showed a decrease in average FBG, systolic blood pressure (SBP), low density lipoprotein cholesterol (LDL-C) by 47.3 mg/dL, 22.6mmHg and 31.4mg/dL, while high density lipoprotein cholesterol (HDL-C) and estimated glomerular filtration rate (eGFR) exhibited significant increase by 13.4 mg/dL and 11.5 ml/min/1.73m2 respectively (p<0.0001). In addition, diastolic blood pressure, lipid values, kidney function parameters, and liver function parameters showed significant decrease in post intervention compared to pre-intervention (p<0.05). Medication adherence of the patients increased significantly at 6-month follow-up (p<0.001).

**Conclusion:**

These results also suggest the benefits of integrating clinical pharmacist services in multidisciplinary healthcare teams and diabetes management in Ethiopia.

## Introduction

Diabetes Mellitus (DM) is a group of metabolic diseases characterized by the occurrence of persistent hyperglycemia due to deficiency in insulin secretion, insulin action, or both 1, 2.

Two-to-three-fold increased risk of cardiovascular disease is associated with type 2 diabetes mellitus. Furthermore, the long-term complications of diabetes are associated with high morbidity, high cost, and decreased quality of life. The factors observed in poor glycemic control include poverty, noncompliance, lack of knowledge, and poor follow- ups 3. Poor adherence results in the worsening of glucose control and increases the hospital admissions of patients due to diabetes complications 4.

In Ethiopia, about 1.8 million adults were living with diabetes 5, 6. The WHO report indicated that the prevalence of diabetes in Ethiopia increased from 0.5% in 1980 through to 3.8% in 2014 6 to 5.2% in 2017(2). A few other studies in Ethiopia report the prevalence of diabetes mellitus in a range from 0.5% to 6.5% 7–9. In Hosanna town, a community-based study done in 2017 shows that about 5.7% of adults 18 years and above live with diabetes 10.

Pharmacists can play an important role in diabetes treatment by helping patients improve their chances of reaching therapeutic and lifestyle goals. As experts in drug therapy, drug selection, and patient education, pharmacists can be worthy or notable additions to the multidisciplinary health care team, contributing to better care for patients 11, 12.

Although global evidence supports the positive impact of pharmaceutical care interventions among patients with diabetes, it is unknown whether such interventions would improve patient outcomes in a comprehensive specialized hospital setting in Ethiopia.

Pharmacists' roles are largely limited to traditional dispensing and medication reconciliation upon limited physician referrals within a comprehensive specialized hospital in Ethiopia. Moreover, pharmacist-led services and collaborative care models change among distinctive nations and settings. Even though many studies are available on the PC service in different countries, no study exists about this service in diabetes patients of Ethiopia. To our knowledge, there were no studies conducted in Ethiopia to document and assess the impact of a pharmacist's care in the context of a pharmaceutical care service (PCS) on the clinical outcomes of diabetes mellitus. Thus, there is a need to demonstrate the benefits of a pharmaceutical care service (PCS) in an Ethiopia setting using a pre-post-interventional design.

## Aim of the study

This aim of this study was to evaluate the impact of pharmaceutical care interventions on health-related clinical outcomes, and medication adherence in patients with diabetes receiving care in a chronic follow-up clinic over a 6-month period in WUNEMMCSH, Ethiopia.

## Ethical approval

The study was approved by ethical committee of WUNEMMCSH medical and research ethics, reference number: 0078/2020 and conducted in accordance to the Declaration of Helsinki. The informed consent was obtained from all the subjects. The consent was moreover obtained from the study participants to publish the study comes.

## Methods

### Study design

A single cantered, pre-post interventional study was carried out at WUNEMMCSH, Hosanna. Data were collected over a period of 6 months with two follow ups in total, each follow up after every three months.

### Sample size

Sample size was estimated based on the prevalence of diabetes in Hosanna town 10, i.e., 5.7%, using Daniel formula^13^ where the prevalence of diabetes in Hosanna town is 5.7%, so P=0.057, while Z = 1.96 (for 95% level of confidence) and d = 0.05.



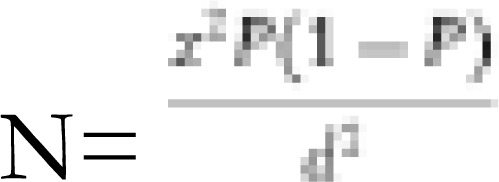



The calculated sample size was found to be 83. However, the data of 100 patients were collected to compensate for the missing or dropouts.

During the three months recruitment periods, patients who had an appointment at the diabetes clinic of WUNEMMCSH were made the sampling frame, and the sampling fraction was calculated.

The average 20 daily attendees were used for calculation of constant (k). The total sample size (100) was divided by the number of days the clinic provide service within three months (24days) of the recruitment period to get the estimated sample of participants per day. Based on this calculation about 4 patients were sampled each day. This was made for the purpose of participant distribution all through the review time frame for better representativeness. A systematic random sampling technique was used based on listt of patients' appointment record by calculating sampling interval as K = N/n (where N (20) average number of patients per day; n (4) is sample to be taken per day). Then participant's medical card numbers (ID) were taken every fifth interval for comprehensive chart review. A colored marker was posted on the patient chart to identify easily during follow up and to avoid double recruitments. Atherogenic index was calculated by using formula = log (TG/HDL-C).

### Study setting

The study was carried out at WUNEMMCSH, a 400-bed facility; it is the referral centre in the area with multiple specialized clinics in southern Ethiopia. The diabetes clinic at the WUNEMMCSH provides usual care services to more than 6,000 diabetic patients on an outpatient level annually with regular clinic visits with an average of about 150 patients per week depending on the glycaemic control for each patient.

### Patient recruitment

Un-controlled T2DM patients (FBG>130mg/dl) were given with details on the intervention conduct and operational methods by research clinical pharmacist. The participants were recruited from March 1- August 30, 2020.

### First phase: Pre-intervention

#### Baseline data collection and evaluation

Patient baseline evaluation was done for the participants that included demographics, physical assessment parameters and laboratory measurements.

Baseline information for each patient was collected by the research clinical pharmacist using a custom-designed questionnaire, medical charts, and health centre computer. The collected data included socio-demographics characteristics, clinical characteristics, and medication characteristics of the participants. The patient also completed Morisky-Green Levine (MGL) medication adherence Scale.

Except for demographic data, baseline data collection measures including all laboratory measurements and questionnaire data were repeated by the research pharmacist (BB) with the assistance of data collectors during scheduled diabetes clinic visits i.e., 3-months after the initial visit for each patient.

#### Missing data or dropouts

**Figure 1:**
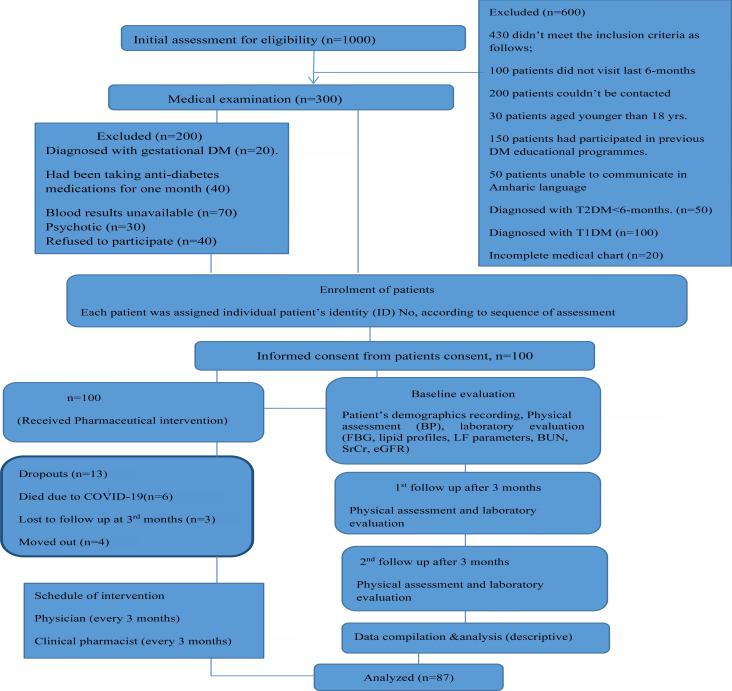
Flow diagram of selection of patients based on pre-defined inclusion and exclusion criteria.

There were 13 drop outs over the study period (6 died due to COVID-19, 3 lost to follow up and 4 moved out). Thus, the final analysis was done on 87 patients over the study period (Fig.1).

#### Study population

Out of total 300 eligible uncontrolled T2DM patients enrolled in the study, 200 failed to provide the informed consent. Uncontrolled diabetes was confirmed by FBG levels above 130mg/dL, according to ADA guidelines^14^. Thus, only 100 subjects were considered for baseline evaluation (Fig.1) based on the study's inclusion and exclusion criteria.

#### Inclusion criteria

Uncontrolled T2DM patients, above 18 years of age, must be visiting the clinic for the last 6 months, taking anti-diabetes medications for at least three months, diagnosed with T2DM for at least 6 months previously, and willing to participate in the study.

#### Exclusion criteria

Missing visits in the previous six months, patients below 18 years of age, participated in DM educational programs in the last 3 months, cognitive impairment, not willing to participate, unable to communicate, and understand Amharic language were excluded.

#### Research progressions

The baseline evaluation of participants was performed using data collection form. The initial education and counselling were about disease, therapy, life style modifications, and self-monitoring of blood glucose. Patients were asked to visit each 4 weeks for the evaluation of pharmacological and non-pharmacological needs, whereas the routine schedule take after up was each three months. Patients were informed about upcoming visits through short message service (sms). The findings were documented and assessed to prepare individualized pharmaceutical care intervention.

### Second phase: post-intervention

#### Pharmaceutical care work plan

A comprehensive pharmaceutical care plan for the patients was designed by intervening clinical pharmacist. On every visit after 4 weeks, the intervening pharmacist assessed the patient's individual needs for the modification of PC based on patient's response toward intervention and self-monitoring record of blood glucose and blood pressure measurements. This was done in the form of progress notes followed by discussions with the study physicians for treatment modifications, if any.

#### Physician cooperation

After baseline evaluation and documentation of patient's clinical and laboratory parameters, at each take after up, progressive revisions in pharmacological needs, or intensification of existing therapy at each follow up taking into account patient's current medications, clinical and Lab data were suggested in consultation with study doctor on the same day, majorly by advance notes and after an isolated dialog in physician's room or when necessary, in the patient's presence. Patients were given with directives on the prescription or on a separate page.

#### Patient-pharmacist interaction

Patient-pharmacist interaction occurred in a separate room designated for patient education and counselling i.e., left after the patient-physician encounter and pertinent laboratory tests. The average time of sessions was 30–45 min, but time varied depending upon the patient's needs and issues. Within this session pharmacists performed case assessments, made advance notes, and create intervention plan to be sent to the doctor. A comprehensive pharmaceutical care plan was planned for each patient by understanding patient's non-pharmacological and pharmacological needs. A detailed description of pharmacist intervention is given below in pharmaceutical care intervention section.

#### Description of Pharmaceutical care interventions

Following baseline assessment, the clinical pharmacist ensured a patient visit every month for the assessment of pharmacological and non-pharmacological needs, while the routine follow-up was every three months.

After patient-physician encounter at each follow up, patients were received by the pharmacist in a designated room. The clinical pharmacist interviewed and provided with structured patient education, discussions about type 2 diabetes mellitus, risk for and types of complications from diabetes mellitus, prescribed drug therapy, proper dosage, possible side effects, and simplification of dosage regimens if deemed appropriate, taking account of the latest American Diabetes Association (ADA) recommendations 14 and the importance of medication adherence. The clinical pharmacists also emphasized lifestyle management as follows: patients were encouraged to (a) healthy lifestyle, (b) self-monitoring of glycemic control (SMBG), and (c) medication adherence.

At last, all patients were probed and their understanding was checked. In order to maintain uniformity of intervention, a single clinical pharmacist conducted the baseline and interventional sessions. Study participants were well informed that they can contact anytime if they need any advice regarding their medications and once a phone call is received, it would then be recorded to know any changes and anticipate any challenge in the implementation of the study.

#### Insulin administration and Oral hypoglycaemic agents

Patients were briefed about the time of administration, i.e., with food or 15–30 min prior to meal, dosing plan, conceivable side effects and frequently interacting drugs. Patients were counselled on insulin administration focusing on injection technique, use of syringe, insulin pen and correct ways of administration. Subjects were told all about the finest injection sites, i-e., thighs and guts, and keep in mind to keep rotating the infusion location.

#### Outcome Measures

The outcome measures examined to assess the impact of pharmacist intervention in the management of T2DM are described below as;

#### Primary outcome measure

The change in Fasting plasma glucose levels from baseline to end of the study, measured by taking plasma venous samples and sending them to laboratory centre, WUNEMMCSH, Hosanna.

#### Secondary outcome measures

The change from baseline to post-intervention blood pressure, lipid profiles (LDL-C, HDL-C, TC, and TG), liver function parameters (AST, ALT, and ALP), and kidney function parameters (BUN, SrCr, and eGFR) measured via sphygmomanometer and by sending samples to laboratory centre, WUNEMMCSH, Hosanna and medication adherence. All the laboratory measurements were performed by the responsible specialist, whereas the plasma samples were collected by a trained nurse.

#### Medication Adherence and treatment goals

6-month assessments. This test consists of 4 questions and each of the four items was in a (yes/no) format:

(1) “Have you ever (do you ever) forgotten to take your medicine?” (2) “Are you careless at times about taking your medicine?” (3) “When you feel better, do you sometimes stop taking your medicine?” and (4) “Sometimes, if you feel worse when you take your medicine, do you stop taking your medicine?”

A “yes” was given a score of 1, and a “no” was given a score of 0. Therefore, the total scores varied between 0–4. The adherence scores were calculated, and participants were categorized as: MGL=3–4 represents low adherence, MGL=1–2 represents medium adherence, and MGL=0 represents high adherence. Patients were considered adherent to pharmacotherapy if they answered “to” all 4 questions. If a patient answered “yes” to any question, the patient was considered non-adherent.

Besides, patients were also briefed how to assess episodes of hypoglycaemia and hyperglycaemia and ways to avoid them. Patients were also briefed about the goals of therapy that is necessary to achieve effective glycaemic control, i.e., FPG levels of ≤110 mg/dL. Blood pressure goals for diabetic patients were < 140/90 mmHg, however, pharmacist intervention was started on BP of 130/85 mmHg. For lipid goals, low density lipoprotein (LDL) value of < 100 g/dL was set for diabetic patients, hence LDL value of > 130 mg/dL was considered cut off for beginning statins.

### Statistical Analysis

Data collected at baseline ,3-months, and 6-months assessments were coded and entered into Epi-Info software, version 7 and exported to Statistical Package for Social Sciences (IBM SPSS 21, Armonk, NY) for statistical analysis. Descriptive statistics were utilized to compare frequency distribution patterns of categorical variables. Baseline characteristics were compared using Pearson chi-square, Fisher's exact test, and one way ANOVA. All continuous and discrete variables were reported as mean and standard deviation from their respective means. The outcome measures i.e., blood glucose levels, blood pressure, lipid profiles, liver function parameters, and kidney function parameters were measured at the same time with similar procedures for participants. The averages of outcome variables were computed before intervention and for each follow ups (after intervention). For continuous variables, the normality of data was tested first using the Kolmogorov-Smirnov and Shapiro-Wilk statistical tests. Significance in those tests indicated that the continuous variable was not normally distributed. Normality test results for continuous variables showed non-normal distributions, and Wilcoxon signed rank test were used for non-normally distributed variables. The variations in means of continuous and discrete variables were compared between pre- and post-intervention. The Wilcoxon signed rank test was used to determine the differences between the mean of before and after-intervention of clinical outcome measures. Paired samples T- test was used to evaluate the effect of pharmaceutical care lead service on medication adherence. A P-value of < 0.05 was considered statistically significant.

## Results

### Participants baseline characteristics

Of the 100 patients initially enrolled, 87(87%) completed the follow-up. Patients baseline socio-demographics and clinical characteristics of participants are summarized in Table 1 and Table 2.

**Table 1:** Baseline Socio-demographic characteristics of all participants completed follow-up (n=87)

Variables	N (%)
Age (yrs.), mean (SD)	50.1(11.9)
<50	40(46%)
>50	47(54%)
Gender	
Male	57(65.5%)
Female	30(34.5%)
Religion	
Orthodox	14(16.1%)
Muslim	12(13.8%)
Protestant	59(67.8%)
Others*	2(2.3%)
Residence	
Urban	48(55.2%)
Rural	39(44.8%)
Marital status	
Single	10(11.5%)
Married	77(88.5%)
Education	
Cannot read and write	26(29.9%)
Primary school	27(31%)
secondary school	13(14.9%)
College /University	21(24.1%)
Working status	
Government employed	17(19.5%)
No job/housewife	40(46%)
Private office/self employed	30(34.5%)

Others*; Catholic

**Table 2:** Baseline clinical characteristics of all participants included in analysis (n=87)

Parameters	N (%)
Duration of DM diagnosis (yrs.), mean (SD)	5.2(3.7)
<5	56(64.4%)
6–10	25(28.7%)
11–20	5(5.7%)
>20	1(1.1%)
DM related complication	
Yes	3(3.4%)
No	84(96.6%)
Number of complications	
0	84(96.6%)
1	3(3.4%)
Complications encountered	
Neuropathy	1(1.1%)
Retinopathy	1(1.1%)
Others**	1(1.1%)
Co morbidity	
Yes	33(37.9%)
No	54(62.1%)
Co morbidity encountered	
Hypertension	30(65.5%)
Ischemic heart disease	1(1.1%)
Dyslipidemia	2(2.3%)
Number of concurrent diseases	
0	54(62.1%)
1	32(36.8%)
2	1(1.1%)

Others**; Diabetic keto-acidosis

### Socio demographic characteristics

The age, gender, marital status, religion, education status, residence, and working status are summarized in Table 1. The average age of the participants was 50.1(11.9) years, ranging from 27 to 76 years in the study.

### Clinical characteristics

DM related complications, comorbidities encountered, DM related hospitalization, number of hospitalizations, reason for hospitalization, duration of DM diagnosis, and having a personal glucometer are presented in Table 2.

Data suggested that the average durations of DM diagnosis was 5.2(3.7) years, with 1 to 27 years. Surprisingly, only 11 (11.9%) participants had access to a personal glucometer in the study (Supplementary file 2).

### Medication characteristics

Table 3 summarized the frequency of antidiabetic medications, other medications prescribed, duration of DM treatment, nondrug treatment plans, traditional medication use, and source of medication for participants.

**Table 3:** Medication characteristics of all participants included in analysis (n=87)

Parameters	N (%)
Duration of DM treatment (yrs.), mean (SD)	5.2(3.8)
<5	57(65.5%)
>5	30(34.5%)
Anti-diabetic and other medication prescribed	
Total number of prescribed medications, mean (SD)	2.2(1.2)
<4	81(93.1%)
>4	6(6.9%)
Glucose lowering medication	
Biguanide (metformin)	
Yes	60(69%)
No	27(31%)
Sulfonylurea (Glibenclamide)	
Yes	44(50.6%)
No	43(49.4%)
Insulin	
Yes	30(34.5%)
No	57(665.5%)
Combination therapy (OHA) Metformin +Glibenclamide	43(49.4%)
Monotherapy	
Metformin	17(19.5%)
Glibenclamide	1(1.1%)
Blood pressure–lowering medication	
ACEI	
Enalapril	
Yes	13(14.9%)
No	74(85.1%)
Lisinopril	
Yes	0
No	87(100%)
Diuretics (Thiazide)	
Yes	12(13.8%)
No	75(86.2%)
Calcium channel blocker(nifedipine)	
Yes	17(19.5%)
No	70(80.5%)
Lipid-lowering medication	
Statin (simvastatin)	
Yes	6(6.9%)
No	81(93.1%)
Aspirin	
Yes	3(3.4%)
No	84(96.6%)
Others***	3(3.4%)

Others***; Neurobin, amitriptyline

The mean (SD) durations of DM treatment were 5.2(3.8) years, ranging from 1 to 23 years.

Of all participants in the study, 15(17.2%) participants used traditional medications for treatment purposes and the most commonly used traditional medications were Moringa *(Shiferaw or Stenopetala cuff)* followed by *Anamura (Armagusa or Ajuga intigrifolia Buch-Ham)*. The majority of the study participants, 73(83.9%) paid for their medication (Supplementary file 3).

### Impact on glycemic goals and other targets of diabetes care

As shown in Table 4, 68(78.2%) patients achieved FBG goals of 80–130mg/dL. Conversely, 19 (21.8%) patients have sustained FBG ≥130mg/dL levels. At final follow up, 83.9% patients achieved SBP goals of < 140 mmHg. While, 16.1% patients achieved SBP goal of ≥140 mmHg. As for DBP, compared to 73.6% subjects at baseline, 6.9% subjects achieved ≥90 mmHg at post-baseline.

**Table 4:** Percentages of patients achieving glycemic, blood pressure and lipid value goals

Outcome measures	Pre-intervention	Post-intervention	
	baseline	3rd month	6th month
Glycemic Goals (FBG)			
% of patients achieving goal <80mg/dL	a0	0	0
% of patients achieving goal 80– 130mg/dL	0	5(5.7%)	68(78.1%)
% of patients l >130mg/dl	87(100%)	82(94.3%)	19(21.8%)
Blood pressure Goals			
% of patients with SBP <140mmHg	8(9.2%)	31(35.6%)	73(83.9%)
% of patients with SBP >140mmHg	79(90.8%)	56(64.4%)	14(16.1%)
% of patients with DBP <90mmHg	23(26.4%)	67(77%)	81(93.1%)
% of patients with DBP >90mmHg	64(73.6%)	20(23%)	6(6.9%)
Lipid profile Goals			
% of patients with LDL <100mg/dL	26(29.9%)	46(52.9%)	75(86.2%)
% of patients with LDL >100mg/dL	61(70.1%)	41(47.1%)	12(13.8%)
% of patients with HDL <40mg/dL	84(96.6%)	46(52.9%)	4(4.6%)
% of patients with HDL >40mg/dL	3(3.4%)	41(47.1%)	83(95.4%)

**Abbreviations:** FBG: - fasting blood glucose, SBP: - Systolic blood pressure, DBP: - Diastolic blood pressure, LDL-C: - Low density lipid cholesterol, HDL-C: - High density Lipid cholesterol.

a Note: Inclusion criteria of study was FBG >130mg/dL i.e., patients with uncontrolled diabetes mellitus.

At post-intervention, 86.2% subjects achieved LDL-C goals of < 100 mg/dL. As for HDL-C, compared to 3.4 % subjects before intervention, 95.4% subjects achieved ≥40mg/dL in post-intervention (Table 4)

### Clinical outcome measures

There weren't difficulties in the data collection instruments during the study. Changes in key clinical parameters over 6 months are shown in Table 5, including statistically significant differences in mean (SD) for the clinical outcomes measured at baseline, 3-months and 6-months. In pre-post intervention, from baseline (B) to 1^st^, and final follow up (F), significant differences were observed in process outcome measures starting from 1st follow up till final follow up. Notables ones included, FBG (B; 172.6(20.2), 1^st^; 149.5(12.8), F; 125.3(13.2), p < 0.0001), SBP (B; 155.5(10.2), 1^st^; 144.3(8.1), F; 132.8(9.5), p<0.0001), DBP (B; 95.9(8.2), 1^st^; 88.9(6.1), F; 81.6(6.4), p< 0.0001), TG (B; 151.4(29.2), 1^st^;143.6(24.1), F;126.4(18.9), p<0.0001), TC (B; 166.5(31.5), 1^st^; 150.9(24.4), F; 135.3(27.2), p < 0.0001), ALT (B; 27.9(8.4), 1st; 22.4(7.5), F; 16.7(6.5), p < 0.0001), AST (B; 34.9(6.6), 1^st^; 2.6(6.5), F; 16.9(7.2), p < 0.0001), BUN (B; 20.7(3.1), 1^st^; 15.9(3.5), F; 11.1(3.8), p < 0.0001), and SrCr (B; 0.92(0.2), 1st; 0.73(0.1), F; 0.56(0.1), p < 0.0001), while eGFR (B; 83.6(10.6), 1st; 89.1(10.2), F; 95.4(9.4), p < 0.0001) exhibited a significant increase. When we calculated atherogenic index of plasma (AIP) at 6 months follow up it is 0.08, which is in low risk as it is AIP<0.11 and significant difference were observed in AIP at 6 months post-intervention from baseline. This is also consistent with decreased TG level, increased HDL level, improving gylcemic control and finally absence of clinically significant atherosclerotic cardiovascular disease (stroke, coronary heart disease, PAD) on patients being followed during our study period and to compare ratio with direct small dense LDL level (LDL sub fraction), we did not have electrophoresis in our study area (Table 5).

**Table 5:** Clinical outcome measures for mean scores and Wilcoxon signed rank test among adult diabetic patients.

Outcome parameters	Mean (SD)				P-values
		Follow up ev ery 3 months			
	Pre- intervention	Post-intervention			
	Baseline	3-month	End of study (6-month)	MD	
FBG	172.6(20.2)	149.5(12.8)	125.3(13.2)	-47.3	<0.0001
SBP	155.5(10.2)	144.3(8.1)	132.8(9.5)	-22.6	<0.0001
DBP	95.9(8.2)	88.9(6.1)	81.6(6.4)	-14.3	<0.0001
LDL-C	112(18.9)	96.4(16.3)	80.6(16)	-31.4	<0.0001
HDL-C	32.5(4.5)	39.1(3.8)	45.8(4.3)	13.4	<0.0001
TG	151.4(29.2)	143.6(24.1)	126.4(18.9)	-24.9	<0.0001
TC	166.5(31.5)	150.9(24.4)	135.3(27.2)	-31.3	<0.0001
ALT	27.9(8.4)	22.4(7.5)	16.7(6.5)	-11.2	<0.0001
AST	34.9(6.6)	25.6(6.5)	16.9(7.2)	-18.1	<0.0001
ALP	140.9(14.7)	128.1(17.3)	112.8(19.6)	-28.1	<0.0001
BUN	20.7(3.1)	15.9(3.5)	11.1(3.8)	-9.6	<0.0001
SrCr.	0.92(0.2)	0.73(0.1)	0.56(0.1)	-0.4	<0.0001
eGFR (ml/min/1.73m^2^)	83.6(10.6)	89.1(10.2)	95.0(9.4)	11.5	<0.0001
AIP	0.3	0.21	0.08	-0.22	<0.001

**Abbreviations:** FBG:- Fasting blood glucose, SBP:- Systolic blood pressure, DBP:- Diastolic blood pressure, LDL-C:-Low density lipoprotein cholesterol, HDL-C:- High density Lipoprotein cholesterol, TCG:- Triglycerides, TC:- Total cholesterol, ALT:- Alanine transaminase, AST:- Aspartate transaminase, ALP:- Alkaline phosphatase, BUN:- Blood urea nitrogen, SrCr:-Serum creatinine, eGFR:- Estimated Glomerular Filtration rate, SD:- Standard deviation, MD:-mean difference between end of study and baseline, AIP:-Atherogenic index of plasma, P-value:- When baseline values and 6 month values are compared.

### Medication adherence to the prescribed medications

According to the Morisky-Green test at baseline, (23%) patients were non-adherent. Morisky Green test scores significantly increased (t-value of 5.322; P<0.001) in post-intervention which indicated that the patients achieved more adherence and better control of glycemic control when compared to baseline. There was significant difference between the levels of adherence at baseline and 6 months post-baseline (Table 6).

**Table 6:** Pre- post intervention changes in Medication adherence rate among adult diabetic patients

Evaluation	Level of adherence		Mean (SD)	t	P value
Baseline (pre- intervention)	Low adherence	20(23%)	1.22(0.44)	5.322	<0.001
	Medium adherence	66(75.9%)			
	High adherence	1(1.1%)			
Final follow-up (post- intervention)	Low adherence	0	0.93(0.25)		
	Medium adherence	81(93.1%)			
	High adherence	6(6.9%)			

## Discussion

To the best of our understanding, this is the first single centred, pre-post interventional study developed in a comprehensive specialized hospital in a developing country like Ethiopia to evaluate the impact of PCS on clinical outcomes and medication adherence in type 2 diabetes mellitus at WUNEMMCSH. The role of clinical pharmacy services are new concepts in Ethiopia and no study has evaluated the effects of PCS on patients with diabetes mellitus in Ethiopia.

In the present study, pharmacist's intervention regarding therapy, lifestyle change management resulted in significant improvements in several process outcome measures, such as glycaemic, blood pressure, lipid controls, liver function parameters along with kidney function parameters in comparison to baseline. Moreover, this pilot study has shown that statistically significant improvements in medication adherence at the 6-month post intervention. In present study, we observed a reduction in FBG levels over the 6-months post intervention and this finding is consistent with other findings; Jarab et al. 16 reported a significant decrease in FBG in patients at the end of a 6-month follow-up period. In Brazil, Mourao et al. showed a significant reduction in FBG over 6 months 17. In study by Wishah RA et al. showed a significant mean reduction in FBG (53mg/dl) *(p<0.05)* over 6 months 18. In India, Choudhary et al. showing a significant decrease in FBG *(p<0.001)* 19. In Emirates University (UAE), Al Mazroui et al. reported a significant decrease in FBG patients who received pharmaceutical care intervention at the end of a 12-month follow-up period *(p<0.001)* 20. These results provide clinical evidence that pharmaceutical care features a positive role in T2DM management and suggest that routine participation of clinical pharmacists in medical teams for outpatients is of high therapeutic value 21.

In our study, the post-intervention showed a significant decrease in SBP (*p <0.0001)* and DBP *(p<0.0001)* compared with baseline values. An important finding was that significantly more patients in the post-intervention (83.9% for SBP and 93.1% for DBP) than baseline (9.2% for SBP and 26.4% for DBP) achieved the ADA target goals (< 140/90 mmHg) 14. Our study findings are consistent with other studies from Korcegez et al. 22 and Shao et al. 21 who reported a significant reduction in SBP *(p=0.01)* and DBP (p=0.04) and the percentage of patients who achieved the target goal of SBP and DBP in line with ADA goals.

Consistent with findings from the current study, earlier studies found that a pharmacist-based management program for patients with T2DM was associated with a significant reduction in blood pressure; Obreli-Neto et al.^23^, Jarab et al., 16, Javaid et al. 24, Ali M et al. 25, Al Mazroui et al.^20^, Abdulrhim et al.^26^, Clifford et al.^27^ and Shao et al. 21 which showed that better blood pressure control and that the pharmacist education sessions and follow up calls proved beneficial in reducing mean SBP and DBP levels significantly.

According to a literature report, a blood pressure increase of 10 mmHg could increase the chance of cardiovascular events by 20% 28. We found that compared to baseline, in the 6-month post intervention there was almost similar reduction in SBP in both males (14.3 mmHg) and females (13.4 mmHg) corroborating previous report that pharmacist intervention resulted in significant improvements in SBP and DBP 20. Thus, it is reasonable to deduce that pharmacist intervention may contribute in averting the chance of cardiovascular events.

With respect to the impact on other targets of diabetes care compared to baseline, beginning from the first take after up, i-e., after 3 months the outcome measures were significantly improved at 6-month post- intervention such as lipid values.

Similar to our findings, numbers of previously reported studies have suggested that pharmacist managed diabetes mellitus care could improve LDL-C, HDL-C, TG and cholesterol levels 16, 23–25, 29–32. The proportion of patients who achieved target LDL-C values (<100mg/dl) and HDL-C (40mg/dL) in our study is in agreement with the study reported by Ali et al. 25, Mourao et al. 17, Wishah et al. 18, Al Mazroui et al., 20 and Hening et al., 33.

Analysis of UKPDS data by Turner et al. indicated that the risk of either angina pectoris or myocardial infarction increases by 1.57 for every 1 mmol/L increase in LDL-C level, and patients with LDL-C levels higher than 3.9 mmol/L were 2.3 times as likely to develop coronary artery disease than those with LDL-C levels less than 3 mmol/L 34.

The significant improvement in lipid values observed in the present study could be due to the clinical pharmacist input and the significant increase in the number of intervention patients who were prescribed statin therapy when compared with baseline at the 6-months assessment. In our study, the improved adherence to medication and education about lifestyle behaviours, particular related to food intake, may have contributed to improving lipid control positively.

The significant improvement in liver function profiles (ALT, AST, and ALP) and kidney function (SrCr and eGFR) in patients at the end of the present study is consistent with findings from earlier research; Javaid et al. 24 reported that a pharmacist-led diabetes service was effective at improving liver function and kidney function tests (SrCr and eGFR). However, there is ample evidence of significant improvement in liver function along with glycemic control after pharmacist intervention 35, 36.

The significant improvement in liver function could be due to the input of pharmaceutical care and improved medication adherence for both antidiabetic medications. Liver enzymes are not the only markers of liver problem, which in fact helps in understanding the severity of diabetes. Elevated serum aminotransferase level; Aspartate aminotransferase (AST), alanine aminotransferase (ALT), and γ-glutamyl transferase (GGT) were commonly observed in diabetes 37. A recent report shows a significant association of increased ALT and AST with insulin resistance, T2DM, and metabolic syndrome 37, 38.

Data to support the impact of pharmacist intervention on liver function in patients with diabetes are limited and warrant further investigation.

Even though diabetes is one of the major public health problems in Ethiopia, there are highly limited documented and updated articles on the association of diabetes with hepatic injury/dysfunction.

According to these diverse results from different studies, the effectiveness of pharmaceutical care in the control of blood glucose, blood pressure, lipid levels, and organ function tests seems to depend on patient behaviours and characteristics, as well as the study design, intervention period, and the different features of the health system where the study was conducted.

We found that compared to baseline, in the 6-month post-intervention patients who received the clinical pharmacy service in the present study demonstrated significantly better self-reported medication adherence, corroborating earlier researches, Mazroui et al. 10, Erku, et al. 39, Korcegez et al., 22 and Jarab et al., 16. Wishah et al. 18, Obreli-Neto et al., 23 and Chung et al., 32 reported that pharmacist intervention resulted in significant improvements in medication adherence.

The finding is similar to previous studies demonstrating the effect of pharmacist interventions to improve medication adherence 21, 31, 40–45.

Within this study, discussions with the pharmacist empowered the development and foundation of much better patient-pharmacist relationship. These consultations expanded the believe of the participants and consequently may have contributed to medication adherence other studies also report that good communication between participants and health care providers leads to favourable and improved medication adherence among people with T2DM 46.

This suggests that the counselling and motivation given by the clinical pharmacist to the patients regarding the appropriate use of medications has led to an increased level of adherence and statistically significant improvement in the follow-up visit.

## Study limitations

Several factors limit the interpretation of our study findings. This study highlights an area of pharmacy practice where there is a lack of literature in Ethiopia

Another limitation of the study was the absence of a control group. The current study was pre-post interventional. To confirm the current finding and reduce the bias, in future a randomized controlled trials (RCTs) shall be undertaken and conducting an RCT is important.

It was conducted in the era of COVID-19 pandemic which was our study follow-up period, so our patients may not have been visiting the scheduled appointment because of the locked down in the country during the study period.

The study is a single centre study that might not allow making generalization to the whole population, so our patients may not have been completely representative of the general diabetic population.

The absence of a gold standard method to measure adherence (we used a self-reported Morisky medication adherence measurement, which is usually associated with recall and social desirability bias) and the inability to access HbA1c measurements in our setting complicated assessment of the interventions provided.

In addition, the intervention period is relatively short (six months) and longer follow-up is needed to determine if the short-term outcomes are sustained by clinical pharmacist interventions.

Regarding TG/HDL ratio as surrogate marker of small dense LDL was not done due to difficulty of comparing TG/HDL ratio with the quantification of small dense LDL (LDL sub fraction) and insulin resistance as LDL sub fraction is done by electrophoresis but it is not available in our set up.

In addition, insulin resistance needs fasting plasma insulin (FPI) level determination by Chemiluminescence immune Assay (CLIA) which is not available in our set up. Indeed, with the over impediments, this study has critical suggestions for moving forward the inclusion of pharmacists in patient care and health advancement in Ethiopia. However, being the first study in this field could open the door for future studies in this important field. Therefore, replication of this study among a larger sample size from many healthcare settings and conducted over a period of follow-up longer than 6 months is recommended for future research.

## Conclusions

The present study demonstrated that pharmaceutical care interventions for patients with type 2 diabetes mellitus improve clinical values, including FBS, blood pressure, lipid profile, and other organ function tests, in addition to Morisky Green Levine medication adherence (MGL). This study demonstrated the vital role of the clinical pharmacist in improving health-related clinical outcomes and medication adherence in patients with type 2 diabetes mellitus in Ethiopia.

## Declarations

### Availability of data and materials

The datasets used and/or analysed during the current study will be made available from the corresponding author on reasonable request.
